# Changes in Apparent Diffusion Coefficient (ADC) in Serial Weekly MRI during Radiotherapy in Patients with Head and Neck Cancer: Results from the PREDICT-HN Study

**DOI:** 10.3390/curroncol29090495

**Published:** 2022-08-31

**Authors:** Sweet Ping Ng, Carlos E. Cardenas, Houda Bahig, Baher Elgohari, Jihong Wang, Jason M. Johnson, Amy C. Moreno, Shalin J. Shah, Adam S. Garden, Jack Phan, G. Brandon Gunn, Steven J. Frank, Yao Ding, Lumine Na, Ying Yuan, Diana Urbauer, Abdallah S. R. Mohamed, David I. Rosenthal, William H. Morrison, Michael P. MacManus, Clifton D. Fuller

**Affiliations:** 1 Department of Radiation Oncology, The University of Texas MD Anderson Cancer Center, Houston, TX 77098, USA; 2 Department of Radiation Oncology, Olivia Newton-John Cancer and Wellness Centre, Austin Health, Melbourne, VIC 3071, Australia; 3 Department of Radiation Oncology, Peter MacCallum Cancer Centre, Melbourne, VIC 3000, Australia; 4 Department of Radiation Physics, The University of Texas MD Anderson Cancer Center, Houston, TX 77098, USA; 5 Department of Radiation Oncology, Centre Hospitalier de l’Université de Montréal, Montreal, QC H2X 3E4, Canada; 6 Department of Clinical Oncology and Nuclear Medicine, Mansoura University, Mansoura 35516, Egypt; 7 Department of Diagnostic Radiology, The University of Texas MD Anderson Cancer Center, Houston, TX 77098, USA; 8 Department of Biostatistics, Peter MacCallum Cancer Centre, Melbourne, VIC 3000, Australia; 9 Department of Biostatistics, The University of Texas MD Anderson Cancer Center, Houston, TX 77098, USA

**Keywords:** apparent diffusion coefficient, head and neck, radiotherapy, gross tumour volume

## Abstract

*Background:* The PREDICT-HN study aimed to systematically assess the kinetics of imaging MR biomarkers during head and neck radiotherapy. *Methods:* Patients with intact squamous cell carcinoma of the head and neck were enrolled. Pre-, during, and post-treatment MRI were obtained. Serial GTV and ADC measurements were recorded. The correlation between each feature and the GTV was calculated using Spearman’s correlation coefficient. The linear mixed model was used to evaluate the change in GTV over time. Results: A total of 41 patients completed the study. The majority (76%) had oropharyngeal cancer. A total of 36 patients had intact primary tumours that can be assessed on MRI, and 31 patients had nodal disease with 46 nodes assessed. Median primary GTV (GTVp) size was 14.1cc. The rate of GTVp shrinkage was highest between pre-treatment and week 4. Patients with T3-T4 tumours had a 3.8-fold decrease in GTVp compared to T1-T2 tumours. The ADC values correlated with residual GTVp. The median nodal volume (GTVn) was 12.4cc. No clinical features were found to correlate with GTVn reduction. The overall change in ADC for GTVn from pre-treatment was significant for 35th–95th percentiles in weeks 1–4 (*p* < 0.001). *Conclusion:* A discrepancy in the trajectory of ADC between primary and nodal sites suggested that they exhibit different treatment responses and should be analysed separately in future studies.

## 1. Introduction

Head and neck squamous cell carcinoma (HNSCC) accounts for approximately 4% of cancer incidence. The majority of localized mucosal HNSCC, particularly within the pharynx, are treated definitively with radiotherapy with/ without chemotherapy. Radiotherapy, although an effective modality of treatment for this group of patients, comes with its own toxicity profile, which at times can significantly affect a patient’s function and/or quality of life later in life, requiring important input from a multidisciplinary team. In recent years, the human papillomavirus (HPV) has been identified as an instigator of oropharyngeal cancer [1]. Although the prevalence of HPV-associated oropharyngeal cancer is on the rise [2], this subgroup of tumours is more radiosensitive and patients with HPV-associated oropharyngeal cancer have improved outcomes compared to those with HPV-negative disease [3,4,5]. Given that this subgroup of patients has a high likelihood of cure with potentially more prolonged survival, there is a push for treatment de-intensification. However, apart from HPV status, there is currently no other biomarker or clinical feature that can help clinicians to better select patients who are suitable or safe for treatment de-intensification, sparing unnecessary treatment-related toxicity. Similarly, for patients with HPV-negative disease who have a higher risk of disease recurrence, there are no validated methods or markers to help clinicians identify patients that may need treatment escalation.

Once a patient embarks on a course of curative-intent radiotherapy, the current workflow does not include granular intra-treatment assessment in the absence of overt clinical disease progression to stratify early responders or non-responders. The PREDICT-HN study was designed to explore the use of serial intra-treatment imaging, using magnetic resonance imaging (MRI), and blood collection to systematically document, describe, and evaluate the kinetics of imaging and blood biomarkers during radiotherapy that could potentially be used to discriminate early responders and non-responders [6]. MRI was selected as the imaging modality of choice in this study as it (1) does not expose the patient to ionizing radiation, (2) provides better soft tissue delineation and therefore improved visualization of the tumour compared to computed tomography (CT), and (3) can perform functional imaging, such as diffusion weighted imaging (DWI), without the need for intravenous contrast. Apparent diffusion coefficient (ADC) is a measure of the diffusion of water molecules from DWI images. There is evidence that ADC is predictive and prognostic in other malignancies [7,8,9,10], and in HNSCC in terms of locoregional failure [11,12]. We have presented a systematic and comprehensive prospective assessment of the kinetics of ADC values in primary and nodal tumours during radiotherapy in patients with HNSCC.

## 2. Materials and Methods

### 2.1. Study Participants

Patients with a pathologic diagnosis of HNSCC scheduled to receive curative-intent radiotherapy were recruited to a prospective observational study (PREDICT-HN study; ClinicalTrials.gov Identifier: NCT03491176) [6]. Eligible patients were of good performance status (ECOG 0–1), had an intact primary or nodal tumour evaluable on MRI, and no clinical evidence of distant metastasis. Patients who had contraindications to MR imaging (pacemaker, neurostimulators, and severe claustrophobia), or had previous radiotherapy to the head and neck region less than 5 years ago were ineligible. This prospective observational protocol was approved by the institutional review board. All patients provided written informed consent prior to study activities.

### 2.2. Radiotherapy Treatment

All patients underwent a standard of care workup and had curative-intent radiotherapy to a total dose of 6996cGy in 212cGy per fraction, 5 fractions per week. During radiotherapy simulation, patients were positioned supine and had a personalized thermoplastic immobilization headrest and mask made. At the treating physician’s discretion, the patient may have an oral stent or bite block. CT images were obtained in the radiotherapy treatment position and images were imported into a treatment planning system for contouring and dosimetry planning. All patients, their relevant imaging and pathology, and contours were reviewed at the radiation oncology quality assurance clinic by a group of expert head and neck radiation oncologists [13]. Patients were treated as per the institutional standard of care protocol.

### 2.3. MR Imaging

MR imaging was obtained within 2 weeks before treatment commencement, weekly during radiotherapy, and at 8 to 12 weeks post-treatment. All imaging was performed in the radiotherapy treatment position with a personalized thermoplastic mask to reduce motion-related artifacts and improve reproducibility [14]. All patients were scanned using the MAGNETOM Aera 1.5T MR scanner (Siemens Healthcare, Germany), with two 4-channel large flex phased-array coils and a 32-channel phased-array spine coil. Table 1 summarises the MRI sequences and parameters used in the study.

### 2.4. Target Delineation

Primary and nodal tumours were delineated on the MRIs as separate structures by two radiation oncologists (S.P.N. and H.B.). An experienced neuroradiologist (J.M.J.) reviewed the delineations for 20% of randomly selected images. Tumours were delineated on the T2-weighted images as they have better anatomical definition and visualization of tumour. The contour was transferred to the corresponding registered ADC map. All contours on the ADC map were checked by S.P.N. to ensure they were within the region of interest and there was no severe image distortion affecting ADC signal value extraction. For those with evaluable primary tumours, two delineations were made on intra-treatment imaging: residual tumour on weekly imaging to obtain residual gross tumour volume, and the area of residual and previous tumour involvement. The area of initial tumour involvement was used to assess ADC value change, as a small residual tumour towards the end of treatment will introduce a bias in measuring the ADC values due to the small volume and imaging pixel size.

### 2.5. Apparent Diffusion Coefficient Values

Raw ADC values of primary and nodal tumours were extracted for every 5th percentile via in-house written code using MATLAB software. This was accomplished by generating ADC value histograms for individual tumours based on the manually contoured volumes.

### 2.6. Statistical Analysis

Missing values were not imputed; where there were missing data for an outcome variable, those records were removed from any formal statistical analysis of that outcome variable (complete case analysis) unless otherwise specified. In tabulations, the numbers of missing observations were provided, but these were not included in the denominator to calculate percentages. Data were split by visit timepoint where applicable. Graphs and descriptive statistics were used to understand the characteristics of gross tumour volume (GTV) and ADC, and the relationship between GTV and ADC.

The correlation between each feature and the GTV was calculated using Spearman’s rank correlation coefficient. The linear mixed model was used to evaluate the change in GTV over time. Factors associated with post-treatment outcome (GTV and ADC) changes from pre-treatment were examined using linear regression adjusted for pre-treatment value, unless otherwise specified. Factors of interest in univariate models included: age at diagnosis, smoking status, ECOG score at pre-radiotherapy, primary site (oropharynx vs. other), T stage (T1-2 vs. T3-4), and N stage (N0-1 vs. N2). Prior to analysis, tests for normality assumption were undertaken, and if the assumption failed, outcomes and/or covariates were naturally log-transformed. All analyses and data manipulations were carried out using SAS 9.4.

This study was designed to enrol 40 patients over a 12-month period. It was expected that 80% (32) would have complete response. Therefore, a 2-sided 90% CI for a difference in tumour size kinetics will extend 0.65 standard units on either size of the difference in sample means. This was calculated using nQuery Advisor.

## 3. Results

### 3.1. Patient Characteristics

Forty-one patients completed the study. The patient, tumour, and treatment characteristics are summarized in Table 2. The median age of the cohort was 59 (range: 41–81) years. The majority were males (90%) and had oropharyngeal cancer (76%). In total, 22 out of 28 patients with oropharyngeal cancer had p16-positive tumours. Most of the cohort had concurrent chemotherapy (80%). Of the 41 patients, 36 had intact primary tumours that could be assessed on MRI, and 31 patients had nodal disease with a total of 46 nodes assessed.

### 3.2. Primary Gross Tumour Volume (GTV) Changes

**Figure 1:**
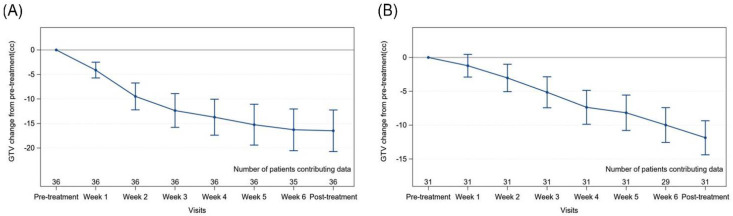
Mean change in primary tumour (**A**) and total nodal volume, (**B**) and from pre-treatment volume to each timepoint with 95% confidence interval. Abbreviation: GTV—gross tumour volume.

**Figure 2:**
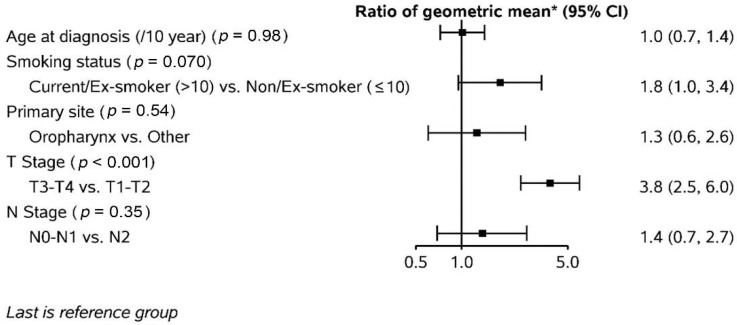
Factors associated with primary tumour volume reduction from pre-treatment to post-treatment. * Ratio of the geometric mean was calculated by log-linear regression models. The GTV reduction was log-transformed in a linear regression model.

Thirty-six primary tumours were assessed. The median primary GTV was 14.1cc (range: 5.1–26.9cc). The average change in primary GTV from pre-treatment volume is illustrated in Figure 1. Overall, the primary GTV change was significant each week (*p* < 0.0001). As shown in Figure 1, the rate of primary GTV shrinkage was highest between pre-treatment and week 4. The average primary GTV reduction per week was 2.3cc. Factors associated with primary GTV reduction from pre-treatment to post-treatment are summarized in Figure 2. Only tumour size (T stage) emerged as a significant factor. Patients with T3-T4 primary tumours had a 3.8-fold (95% CI: 2.5–6.0) decrease in the geometric mean of GTV change at post-treatment compared to those with T1-T2 tumours.

### 3.3. Nodal Gross Tumour Volume (GTV) Changes

Thirty-one (76%) patients had a total of 46 nodes evaluable. The median total nodal volume was 12.4cc (range: 8–21cc). Figure 1 depicts the mean change in nodal volume over time. The change over time was significant (*p* < 0.0001) but when normalized to the pre-treatment volume, only the weekly changes from week 2 onwards are statistically significant (week 2 *p* = 0.002; week 3 to post-treatment *p* < 0.0001). The average nodal volume reduction per week was 1.7cc. No clinical features were found to correlate with nodal volume reduction.

### 3.4. Apparent Diffusion Coefficient (ADC) Changes

**Figure 3:**
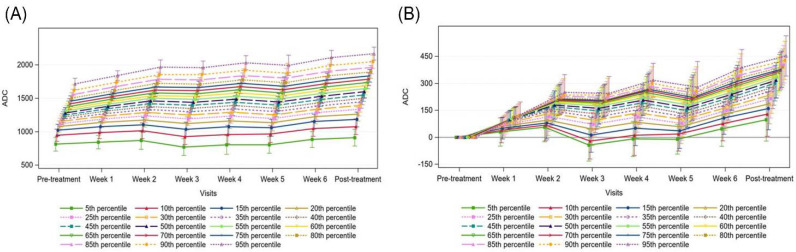
Absolute Apparent Diffusion Coefficient (ADC) measurements, (**A**) and mean ADC change from pre-treatment, (**B**) for the primary tumour, with 95% confidence intervals.

**Figure 4:**
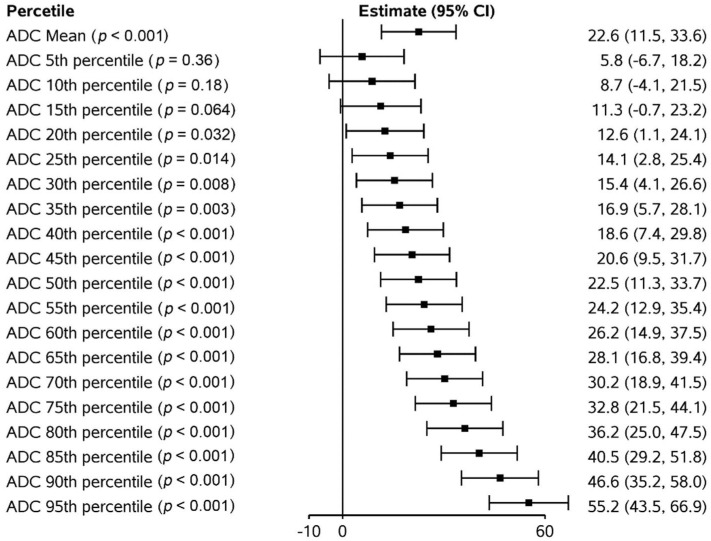
Average change in apparent diffusion coefficient (ADC) values for primary tumour adjusted for pre-treatment ADC.

Figure 3 shows the trajectory of the absolute ADC measurements and ADC values normalized to pre-treatment values for primary tumour sites. Although there was an overall trend of increasing absolute ADC values over time, when normalized to pre-treatment values, we observed a steep increase in ADC values from pre-treatment to week 2, minimal change between weeks 2 and 5, and then a steep increase in ADC values from week 5 to post-treatment. Although there is an overall rise in ADC values across all histogram percentiles (*p* < 0.0001), the ADC change was greater in magnitude in the higher percentiles (Figure 4). As the change from pre-treatment ADC values across timepoints were assessed, we observed that the lower percentiles (5th–40th percentiles) did not meet statistical significance, whilst the change from 45th percentile upwards were significant across all timepoints except for week 1. Similarly, ADC absolute values from the 45th percentile upwards correlated with residual primary tumour volume (all *p* < 0.002, with all above 60th percentiles having *p* values < 0.0001).

**Figure 5:**
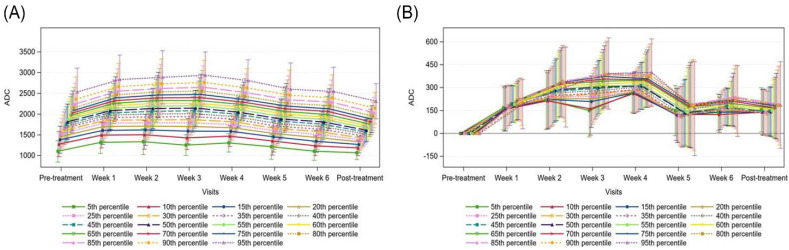
Absolute apparent diffusion coefficient (ADC) measurements (**A**) and mean ADC change from pre-treatment (**B**) for the involved nodes, with 95% confidence intervals.

With regard to involved nodes, Figure 5 illustrates the trajectory of ADC signal values (absolute and normalized to pre-treatment) during the study. When normalized to pre-treatment values, there was a significant rise in ADC values up to week 4. The ADC values then dropped from week 4 to week 5 and remained relatively stable to the post-treatment timepoint. The overall change in ADC values from pre-treatment was significant for 35th to 95th percentiles in weeks 1 to 4 (all *p* < 0.001). In contrast to primary tumour, nodal volume did not correlate with ADC values.

### 3.5. Patient Outcomes

The median follow-up time was 12 (range: 6–22) months. Of the 41 participants, 2 patients had died. At post-treatment, all but three patients achieved complete response. Of the three patients with incomplete response, one died of progressive local disease and two had salvage treatments (neck dissection for persistent nodal disease, confirmed on pathology). During surveillance, eight (20%) patients developed recurrences—four local recurrences, one regional recurrence, one regional and distant recurrences, and two distant diseases. The ADC kinetics of those who had local and regional recurrences are displayed in Supplemental Figure S1.

With a limited number of events thus far, no meaningful correlation between patient outcomes and ADC changes during radiotherapy were made. The survival outcomes of the cohort are summarized in Supplemental Figure S2.

## 4. Discussion

In this prospective observational study, we systematically assessed and quantified the kinetics of ADC and gross tumour volume changes during radiotherapy. By assessing weekly MRIs during radiotherapy, we have shown that the trajectory of primary tumour size and ADC changes is different to that of nodal disease. Firstly, although both primary and nodal tumours exhibit an increase in ADC values over time, the change in ADC values was in a relatively linear fashion in nodes in the first 4 weeks of treatment, whereas the increase in ADC values in primary tumours was more pronounced in the first 2 and final 2 weeks of treatment. Secondly, although the ADC absolute values (above 45th percentile) correlated with residual gross primary tumour volume, no correlation between ADC values and residual nodal volume was observed. Overall, we demonstrated a significant change in ADC values weekly compared to baseline value during radiotherapy. The kinetics of ADC change during treatment is different between primary and nodal tumours, therefore, they should be assessed as separate entities in future studies.

The utility of DWI and ADC as a potential imaging biomarker of response in head and neck cancer radiotherapy has been described in the literature [11]. DWI has been utilized to differentiate malignant versus benign lymph nodes in head and neck [15,16] and in some instances, the histology [17]. The significant increase in ADC values of the tumoural region during and post-treatment had been previously evaluated [11,12,18] with different selections of intra-treatment timepoints. Kim et al. [11], in a cohort of 33 patients, compared ADC values of metastatic nodal mass across three timepoints (pre-treatment, week 1 of therapy, and 2 weeks post-treatment) and showed that the ADC values increased significantly after 1 week of therapy, which is similar to our findings. The increase in ADC in week 1 resulted in more than 80% sensitivity and specificity for predicting complete response. Similarly, Vandecaveye et al. [12] demonstrated that the change in ADC in weeks 2 and 4, rather than the tumour volume change, correlated with 2-year locoregional control. The lack of correlation between tumour volume regression and ADC change, particularly for nodal disease, is reflective of the ability of DWI to detect early tumour microenvironmental changes and malignant cell death before any macroscopic volumetric change is exhibited on imaging, as shown in our study.

Our study demonstrated that the kinetics of tumoural ADC during radiotherapy may provide indication of treatment response and subsequent local and/or regional relapse. Similarly, in a study of 29 patients, Vandecaveye et al. [18] reported that the change in ADC values at 3 weeks after treatment had high positive and negative predictive values for complete response, allowing early evaluation of treatment response compared to standard 3-month post-treatment PET and/or CT evaluation. The current policy of treatment response assessment at 2 to 3 months after radiotherapy is predominantly built upon evidence that CT and/or PET performed during and/or soon after completion of radiotherapy can be challenging to interpret, secondary to the presence of treatment-related inflammation [19,20,21,22]. In contrast, DWI can differentiate treatment-related oedema from residual tumour, allowing its use during radiotherapy and for early treatment response. Head and neck tumours that achieve complete response post-treatment have higher ADC values compared to their corresponding pre-treatment values [11]. ADC can potentially be used during radiotherapy to identify early responders. Early identification of this subgroup of patients may allow for treatment de-escalation and spare unnecessary treatment-related toxicity. In the same vein, identifying those with progressive disease or non-responders allows a change in the patient’s treatment plan, rather than progressing along the same futile treatment pathway.

Previous studies have focused on specific timepoints during radiotherapy to assess changes in ADC values [11,12,18]. There is, however, a lack of standardization of the timing for imaging assessment during radiotherapy. The optimal timing for MRI assessment during treatment remained unknown. Our study was the first to systematically assess and describe the kinetics of weekly ADC change of primary and nodal tumour during radiotherapy. Our study showed that the increase in ADC values during treatment is not linear, and overall, there is a steep increase in ADC value between pre-treatment and week 2, followed by week 5 to post-treatment. The ADC value remained relatively stable between weeks 2 and 5, indicating that future studies may be able to assume that the ADC values between this period are similar. This allows flexibility of MR imaging tumour response assessment during radiotherapy, particularly in institutions with limited MR imaging capacity. Consistent with findings from King et al. [23], where the kinetics of ADC towards the end of treatment may be predictive of locoregional failure, our study highlighted the importance of imaging towards the end of radiotherapy, as an early drop in ADC values is suggestive of incomplete treatment response or subsequent locoregional failure. This early assessment of treatment response, if confirmed with longer follow up, can be a powerful tool for detecting patients at risk of incomplete response or disease relapse. This will allow for an early change in their management plan be it closer surveillance post-treatment, and/or escalation of treatment.

This study had several limitations. Firstly, the sample size was relatively small with 41 patients who completed the study. The predominant reason for the other patients not completing the study was non-compliance with attending weekly imaging due to either social circumstances, and/or patient’s intolerance of an additional MR imaging with immobilization devices in addition to their daily radiotherapy treatments. Secondly, we had a heterogeneous cohort of patients in terms of primary tumour sites. As a first observational study utilizing weekly MR imaging during radiotherapy, we aimed to characterize the kinetics of tumour changes during treatment of patients with head and neck cancer overall. However, our cohort consisted predominantly of patients with oropharyngeal cancer (77%). Therefore, it may be extrapolated that our results may be reflective of those with oropharyngeal squamous cell carcinomas. Thirdly, patients with small primary tumours and small nodes towards the end of treatment were included in the analysis. Small regions of interest could potentially introduce sampling bias secondary to the voxel size of 2 × 2 × 4 mm. The effect of a small sample area may have been reflected in the trajectory of ADC change in nodes, as we observed a reduction in ADC signal after week 4 and this may be due to a volume/sample effect. Lastly, the DWI imaging in this study only used two b-values of 0 and 800, thereby limiting the assessment of intravoxel incoherent motion (IVIM) to further characterize diffusion, versus perfusion of the region of interest. We selected two b-values in this study to reduce the MR scanning time for patients to improve comfort, particularly when they developed acute effects of radiotherapy. Further imaging with multiple b-values can be explored in future studies.

## 5. Conclusions

In conclusion, this study showed that ADC values increase significantly during radiotherapy, particularly within the first 2 weeks of treatment, for both primary tumour and nodal sites. After week 2, a discrepancy in the trajectory of ADC changes was observed between primary and nodal sites suggesting that primary and nodal sites exhibit different treatment responses, which should be analysed separately in future studies. Further follow up and investigations are required to identify the optimal timing of imaging and ADC value assessment during radiotherapy to improve patient risk stratification.

## Figures and Tables

**Table 1:** MRI sequences and parameters obtained during the study [6].

Parameter	T1	T2	DWI (BLADE)
Slice Orientation	Axial	Axial	Axial
Field of View (mm)	256	256	256
Voxel Size (mm)	1 × 1 × 2	1 × 1 × 2	2 × 2 × 4
Recon Voxel Size (mm)	0.5 × 0.5 × 1	0.5 × 0.5 × 2	2 × 2 × 4
Parallel Imaging	No	Yes; Factor 2	No
Slice Number	240	120	25
Fold-over Direction	AP	AP	N/A
Slice Oversampling	100%	No	No
Shim	Auto	Auto	Auto
Scan Mode	3D	M2D	M2D
Technique	GRE	SE	BLADE
Fast Imaging Mode	No	TSE	TSE and EPI
Echoes	1	1	1
Flip Angle(deg)	20	90	90
TR (ms)	7.38	4800	5400
Echo Time (ms)	4.77	80	50
Fat Suppression	No	No	Yes
b-values (s/mm^2^)	N/A	N/A	0.800
NEX	1	1	8
Geometry Correction	3D	2D	No
Echo Train Length	1	15	15
Percent Sampling (%)	80	90	100
Pixel Bandwidth (Hz)	400	300	1220
Scan Duration (min)	6:05	4:48	7:08

**Table 2:** Patient, tumour, and treatment characteristics.

Parameters		*N* = 41	%
Age (median, range)		59 (41–81)	
Sex	Male	37	90
	Female	4	10
Smoking status	Never	17	41
	Ex < 10	6	15
	Ex ≥ 10	10	24
	Current	8	20
ECOG performance status	0	20	49
	1	21	51
Primary tumour site	Oropharynx	28	76
	Larynx	4	11
	Nasopharynx	4	11
	Nasal cavity	1	3
Primary tumour (T) stage (AJCC 7th edition)	T0	3	7
	T1	5	12
	T2	15	37
	T3	7	17
	T4	11	27
Nodal (N) stage (AJCC 7th edition)	N0	5	12
	N1	7	17
	N2 (nasopharynx)	2	5
	N2a	3	7
	N2b	22	54
	N2c	2	5
Radiation modality	Photon	27	66
	Proton	14	34
Concurrent chemotherapy	Yes	33	80
	No	8	20

## Data Availability

The data presented in this study are available on request from the corresponding author. The data are not publicly available due to ethics/privacy reasons.

## Supplementary Materials

The following supporting information can be downloaded at: https://www.mdpi.com/article/10.3390/curroncol29090495/s1, Figure S1: Apparent diffusion coefficient (ADC) kinetics of those who had local and regional recurrences; Figure S2: Overall survival (OS) and progression free survival (PFS) curves for the cohort with 95% confidence interval (95% CI).
