# Supplementary material for: Changes in Apparent Diffusion Coefficient (ADC) in Serial Weekly MRI during Radiotherapy in Patients with Head and Neck Cancer: Results from the PREDICT-HN Study
Source: Curr Oncol. 2022 Aug 31;29(9):6303–13. doi: 10.3390/curroncol29090495 (PMC9498049; doi:10.3390/curroncol29090495)
Supplement: Supplementary file 1 [file curroncol-29-00495-s001.zip › curroncol-1855102-supplementary.pdf]

Supplementary Materials: Baseline Splenic Volume Outweighs Immuno-modulated Size Changes with Regard to Survival Outcome in Patients with Hepatocellular Carcinoma under Immunotherapy

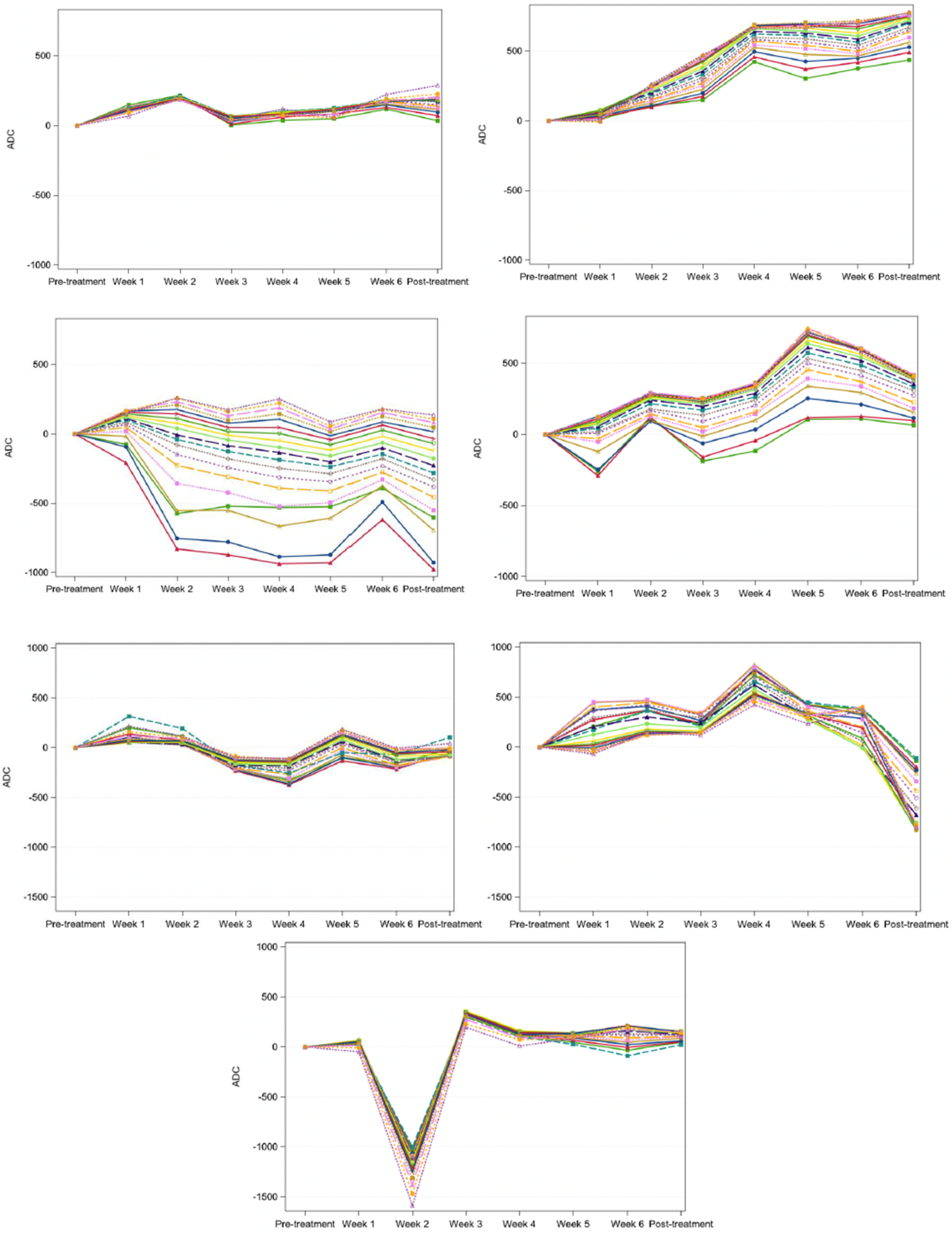

Figure S1. Apparent diffusion coefficient (ADC) kinetics of those who had local and regional recurrences.

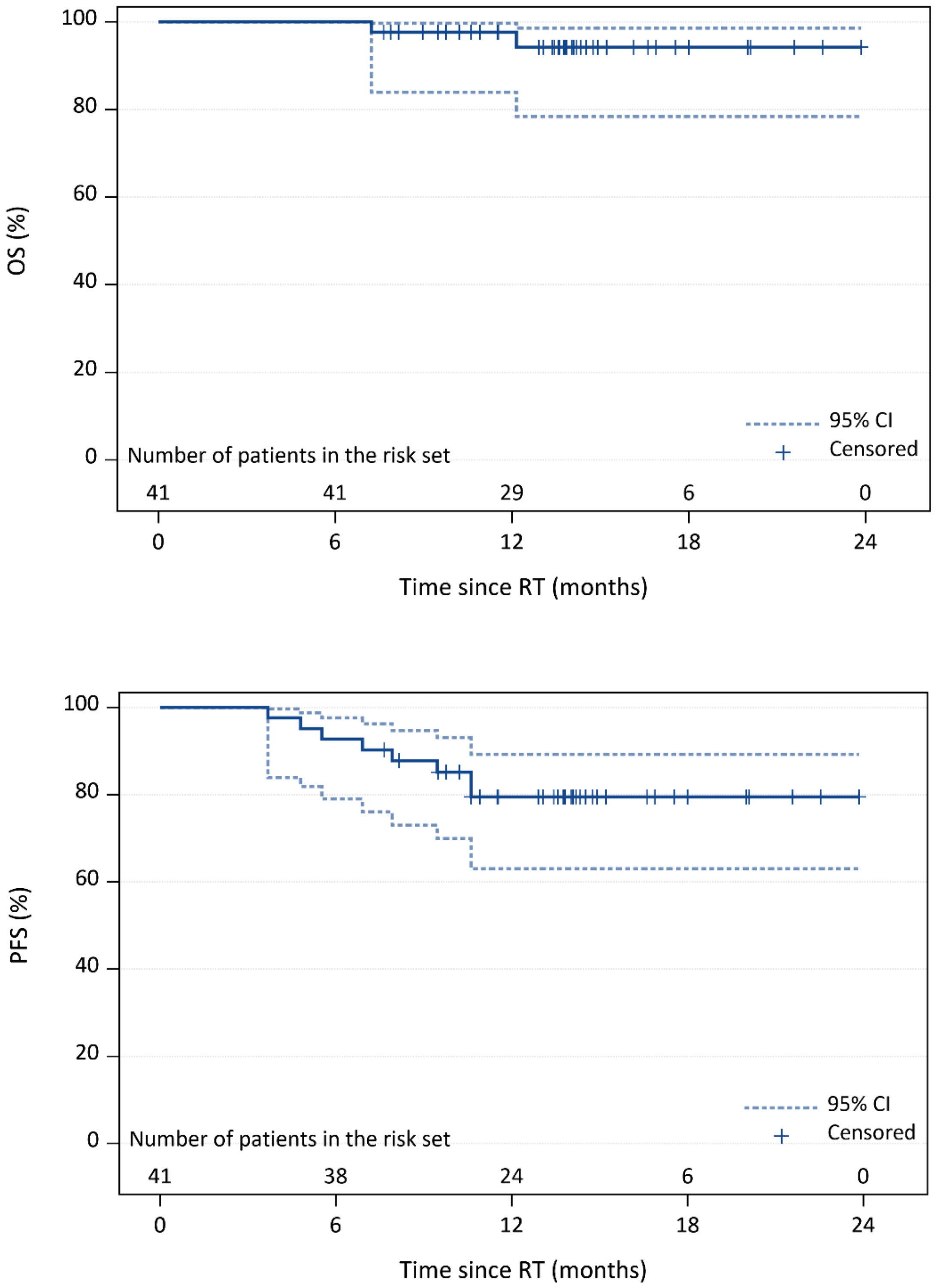

Figure S2. Overall survival (OS) and progression free survival (PFS) curves for the cohort with 95% confidence interval (95% CI)
